# Mechanisms of Motor Adaptation in Reactive Balance Control

**DOI:** 10.1371/journal.pone.0096440

**Published:** 2014-05-08

**Authors:** Torrence D. J. Welch, Lena H. Ting

**Affiliations:** W. H. Coulter Department of Biomedical Engineering, Emory University and Georgia Institute of Technology, Atlanta, Georgia, United States of America; University of California, Merced, United States of America

## Abstract

Balance control must be rapidly modified to provide stability in the face of environmental challenges. Although changes in reactive balance over repeated perturbations have been observed previously, only anticipatory postural adjustments preceding voluntary movements have been studied in the framework of motor adaptation and learning theory. Here, we hypothesized that adaptation occurs in task-level balance control during responses to perturbations due to central changes in the control of both anticipatory and reactive components of balance. Our adaptation paradigm consisted of a Training set of forward support-surface perturbations, a Reversal set of novel countermanding perturbations that reversed direction, and a Washout set identical to the Training set. Adaptation was characterized by a change in a motor variable from the beginning to the end of each set, the presence of aftereffects at the beginning of the Washout set when the novel perturbations were removed, and a return of the variable at the end of the Washout to a level comparable to the end of the Training set. Task-level balance performance was characterized by peak center of mass (CoM) excursion and velocity, which showed adaptive changes with repetitive trials. Only small changes in anticipatory postural control, characterized by body lean and background muscle activity were observed. Adaptation was found in the evoked long-latency muscular response, and also in the sensorimotor transformation mediating that response. Finally, in each set, temporal patterns of muscle activity converged towards an optimum predicted by a trade-off between maximizing motor performance and minimizing muscle activity. Our results suggest that adaptation in balance, as well as other motor tasks, is mediated by altering central sensitivity to perturbations and may be driven by energetic considerations.

## Introduction

Balance control is a fundamental motor task that must be rapidly adapted in the face of a dynamically varying environment, as well as during the performance of concurrent motor activities such as reaching. In standing balance control, the motor goal is to maintain the body center of mass (CoM) in upright postural equilibrium within an unstable gravitational field. To do so requires that the projection of the CoM be maintained over the base of support formed by the feet. Following a perturbation to standing balance, the dynamics of the body are sequentially influenced by anticipatory and reactive mechanisms, which provide interacting and redundant strategies to achieve the motor goal [1], [2]. Because even a small error in motor performance during standing may result in catastrophic failure, such as falling, rapid adaptations to changes in the environment should be expected in balance control. While the anticipatory and reactive components of standing balance have been shown to modulate with environmental context and habituate with repetition, motor adaptation during reactive balance control has not been formally studied in the context of motor learning theory [3], [4].

Motor adaptation has been primarily investigated in voluntary reaching movements where the initial reach direction, reflecting feedforward neural processes, is the primary variable of interest. When reaching in a novel environment such as a curl field or visuomotor rotation, the initial direction of hand movement is modified from trial to trial based on the movement errors in the preceding trial or trials [3], [4]. A hallmark of adaptation is a gradual reduction in errors over repeated trials in the novel environment, with a sustained aftereffect when the novel environment is removed [3], [4]. The presence of aftereffects is critical, demonstrating that a generalized change in an internal model for planning movements is induced, which alters both the motor command executed and the expected sensory feedback resulting from that movement [5]–[10]. This generalization is especially evident when transfer of the adaptation is observed when the movement direction [11], arm configuration [12], [13], movement trajectory [14], [15], or limb [13], [16] differs from that in which the adaptation occurred. Generalization suggests that the adaptation of the initial movement direction reflects a generalized change in the representation of the environment used in planning the motion within the nervous system (i.e., feedforward control), and is not specific to the execution or sensory state of a particular movement [3]. However, the later portions of the response, which may be due to reactive mechanisms in response to trajectory error (i.e., feedback control) have not typically been examined during motor adaptation and are thought to be modulated by a different process [17].

Aftereffects when changing environmental conditions have not been formally demonstrated in reactive responses to perturbations. However, adaptation including aftereffects in anticipatory postural adjustments preceding voluntary movements during standing has been observed [18], [19]. Reactive balance control differs from voluntary movement in that it is inherently a feedback sensorimotor process where muscle activity is activated in direct response to task-level error [20]–[24]. The influence of the sensorimotor feedback response on muscle activity initiates after 50 ms due to spinally-regulated stretch responses in muscles that are lengthened by the perturbation [1], [25]. However, the primary feedback stabilization in response to a perturbation is due to the much larger long-latency automatic postural response, which can be observed as reactive muscle activity after about 100 ms from perturbation onset [25], [26]. This response is mediated by brainstem neural circuits [1] and can be described by a sensorimotor feedback transformation based on the deviations of the CoM kinematics from the desired, upright configuration [20]–[24]. Due to electromechanical delays, the stabilizing effects of this reactive muscle activity on CoM displacement may not be evident for up to 200 ms after the muscle activity is evoked.

With repeated perturbations, a decrease in response amplitude occurs in reactive muscle responses [27]–[29], however it is not known whether this reflects adaptation in the sensorimotor response or changes in the effect of the perturbation on the induced task-level error. Since the effects of the perturbation on the initial CoM motion could also vary across trials, it is possible that the decreased reactive muscle activity is due to a decrease in the sensory stimulus arising from the perturbation itself, which could be affected by anticipatory changes to the amount of lean [30], placement of the feet in a wide or narrow stance [31], and joint stiffness from muscle contraction [2] at the time of perturbation. Although the existence of aftereffects has not been formally studied in reactive balance, the effects of prior perturbation direction and postural conditions on reactive balance responses have been observed [32]–[34]. For example, when presented with the same perturbation, subjects use an ankle strategy when standing on the floor, but a hip strategy when standing on a narrow beam. However, just after standing on the beam, subjects persist in using a hip strategy when perturbed on the floor; the response strategy shifts to an ankle strategy over about seven trials [29].

The sensorimotor feedback transformation we identified in reactive balance control facilitates the interpretation of changes in muscle activity underlying motor adaptation, identifying the sensitivity of the response to a perturbation independent of the magnitude of the error induced by the stimulus. A few studies have examined muscle activity during adaptation where the activity can be reasonably compared, typically during the voluntary initiation of reaching movements [35]–[39] or as an overall amplitude during reaching or locomotor adaptation [39]. However, muscle activity that is evoked due to sensorimotor error is more difficult to study because the amplitude depends directly upon the characteristics of the error, rather than any voluntary central process. Thus, changes in the evoked muscle activity cannot be evaluated independent of the induced error [40]. In balance control, we showed that the time course of muscle activity evoked by a perturbation can be reproduced by a weighted sum of the deviation of the CoM acceleration, velocity, and displacement from the desired upright state [20]–[24]. Three sensorimotor feedback gain parameters thus define the sensitivity of the muscle activity in response to a given error. Therefore, changes in sensorimotor feedback gains indicate a central change in the sensitivity of the nervous system to error, irrespective of its magnitude. Moreover, as changes in the anticipatory aspects of balance control could also affect the degree to which a perturbation induces error in standing balance, examining the sensorimotor transformation is necessary to dissociate the effects of anticipatory versus reactive components of the perturbation response.

Here, our goal was to examine whether adaptation occurs in task-level balance control, as well as in the anticipatory and reactive components contributing to balance control in response to perturbations. We hypothesized that adaptation occurs in task-level balance control during responses to perturbations due to central changes in the control of both anticipatory and reactive components of balance. Our adaptation paradigm consisted of a Training set of identical forward perturbations of the support surface, followed by a Reversal set in which a set of identical countermanding perturbations were introduced. The countermanding perturbation was initially identical to the forward perturbation of the Training set, but then reversed direction at 100 ms, the same approximate latency as the muscular response to the initial perturbation. Finally, a Washout set that was identical to the Training set was presented to test for aftereffects. We predicted that task-level motor performance, as measured by the peak CoM displacement and velocity, would decrease in each set and exhibit aftereffects in the Washout set, as indicated by increased CoM displacement and velocity at the beginning of the set, followed by a return to levels measured at the end of the Training set. As prior adaptation studies have focused on anticipatory or feedforward aspects of motor control, we also predicted that adaptation and aftereffects would be observed in postural lean and muscle activity prior to the perturbation, which could be in part responsible for the changes in motor task performance. We also expected reactive responses to the reversal of perturbation direction to be advanced in time during the Reversal set, when the timing of the perturbation reversal was predictable, as well as a decrease in the magnitude of the responses. Finally, we predicted that these changes in reactive muscle activity would not be entirely accounted for by the decrease in CoM displacement and velocity caused by the perturbation, but by a change in the central sensitivity of the sensorimotor response perturbation. Therefore, we predicted that the feedback gains in the parameters of the sensorimotor transformation would change across each set. Specifically, the magnitude of the feedback gains at the end of the Reversal set would be the same as in the beginning of the Washout set, and then return to levels observed at the end of the Training set.

## Methods

### Ethics Statement

The experimental protocol was approved by both the Georgia Institute of Technology and Emory University Internal Review Boards, and all subjects signed an informed consent form before participation.

### Data Collection

We recruited fifteen healthy subjects (7 male, 8 female), ages 22.5±3.2 years (mean ± standard deviation), from the Georgia Institute of Technology student population who were naïve to postural control studies and had never experienced a perturbation on a moveable platform. Subjects stood upon two force plates installed on a moveable platform that translated in the horizontal plane. Subjects focused vision to a scenic view 4.6 meters away and were instructed to cross their arms at chest level and react naturally to the support surface perturbations, while attempting to keep their feet in place.

**Figure 1:**
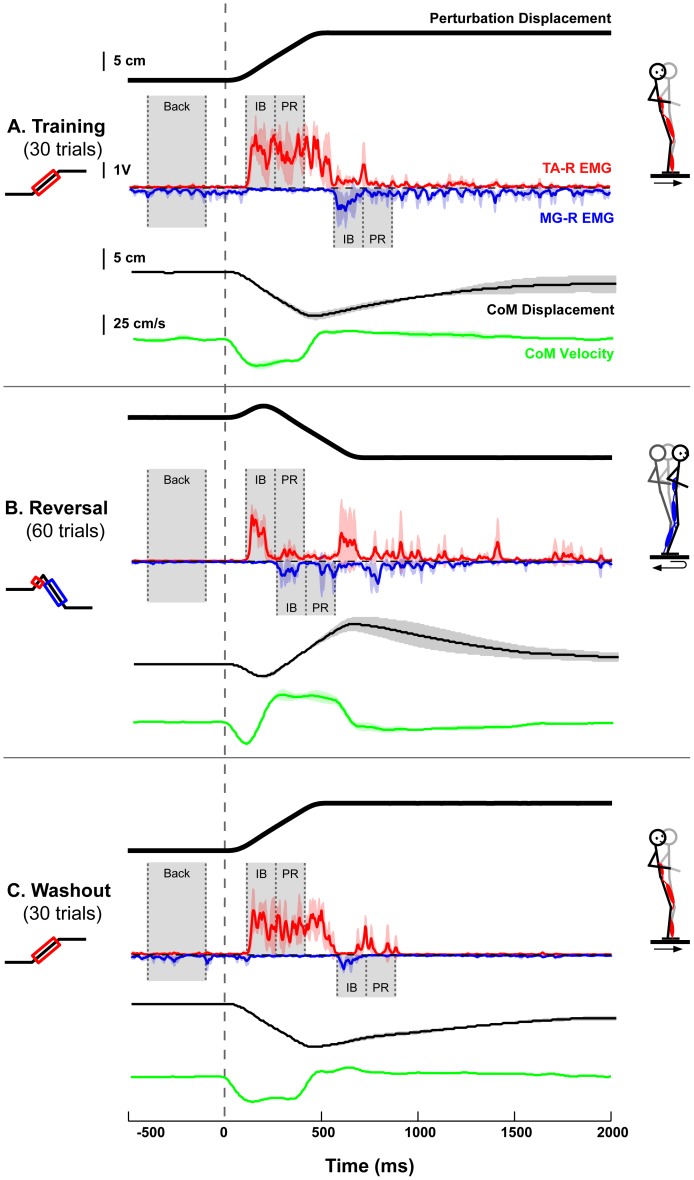
Experimental protocol and example EMG and CoM data. Representative data are illustrated describing the platform displacement and the resulting EMG and CoM kinematics for the administered experimental protocol. **a)** Completely naïve subjects encountered 30 unidirectional forward perturbations (Training). **b)** Then, the platform motion was unexpectedly changed to reverse directions after 100 ms (Reversal). **c)** After 60 reversing perturbations, the platform motion was again unexpectedly changed to a set of 30 unidirectional forward perturbations (Washout). One standard deviation of the mean EMG over three consecutive trials is indicated as a red (TA) or blue (MG) shaded area surrounding the EMG signal. Similar shading is used to indicate one standard deviation of the mean CoM position (black) and velocity (green) over three consecutive trials. Gray shaded areas indicate the 300-ms background period before platform motion used for the statistical evaluation of feedforward changes (Back), and the 150-ms time windows over which feedback muscle response amplitude was evaluated (IB, initial burst; PR, plateau region). Stick figures indicate the direction of platform motion and the intended kinematic motions evoked from each perturbation in the sagittal plane.

A total of 120 sagittal perturbations of the support surface were presented in three sets: Training, Reversal, and Washout. Subjects were not acclimatized to the perturbations prior to the experiment, such that “first responses” [27], [28] were recorded. The Training and Washout sets consisted of 30 unidirectional, forward perturbations (peak acceleration = 0.4g; peak velocity = 35 cm/s; total displacement = 12 cm), which elicited reactive muscle activity in the tibialis anterior (TA) muscle (**Figures 1a and 1c**, red traces). After the Training set, subjects were exposed without warning to the Reversal set, consisting of 60 countermanding [41] perturbations that were initially identical to the forward perturbations of the Training set, but reversed directions due to a negative acceleration applied at 100 ms, which is the expected latency of the TA response to the initial forward platform motion. The reversing perturbations then traveled a total displacement of 12 cm in the backward direction, eliciting reactive muscle activity in the medial gastrocnemius (MG) muscle (**Figure 1b**, blue traces). After the Reversal set, subjects were presented with the Washout set without a break or warning. For all perturbation sets, platform motion was initiated from the same spatial position and, following each perturbation, subjects remained on the platform as it returned to its initial starting position. Time intervals between trials were randomized such that each perturbation was unexpected by the subjects. A minimum of five minutes seated rest was enforced after 60 perturbations to reduce the effects of muscular fatigue; this requirement split the Reversal set into two sets of 30 perturbations.

Data were collected for 3 seconds per trial, including a 500-ms quiet standing period prior to platform motion. Surface electromyograms (EMGs) from right-leg TA and MG muscles, as well as platform acceleration and position, and ground-reaction forces, were collected at 1080 Hz. Raw EMG signals were high-pass filtered at 35 Hz (3rd order zero-lag Butterworth filter), de-meaned, half-wave rectified, and low-pass filtered at 40 Hz (1st order zero-lag Butterworth filter). Platform acceleration and position signals were low-pass filtered at 30 Hz (3rd order zero-lag Butterworth filter). Body segment kinematic trajectories were collected at 120 Hz using a 6-camera Vicon motion analysis system and a custom, bilateral 25-marker set that included head-arms-trunk (HAT), thigh, and shank-foot segments. Center-of-mass motion was calculated from kinematic data as a weighted sum of segmental masses [42].

### Data Analysis

To examine the adaptive modifications in reactive and anticipatory postural control, we computed peak CoM displacement and velocity with respect to the feet, EMG latencies and amplitudes, initial postural lean, and background EMG activity for each trial. Reactive EMG onset timing was determined as the time point following platform motion onset at which the EMG activity exceeded its mean activation level during the quiet background period by two standard deviations. The accuracy of all EMG onset latencies was individually verified by visual inspection. The amplitude of reactive EMG responses was examined during two consecutive 150-ms time bins, beginning at EMG onset, corresponding to the initial burst and plateau regions of muscle activity. Anticipatory changes in the initial CoM displacement from upright (postural lean) and recorded EMG signals during a 300-ms background time period before platform motion (postural tone) were also evaluated (**Figure** **1**, Back).

To evaluate the degree of adaptation, we used paired t-test analysis (α = 0.05) to identify statistically-significant differences between the first and last trials of each set for each measured parameter, and to determine aftereffects between sets. All data were determined to be normally distributed based on the one-sample Kolmogorov-Smirnov test on the differences between the first and last trials (or last and first trials) with a significance level of α = 0.05. We also corrected the results for multiple comparisons based on a Bonferroni correction, accounting for the number of statistical tests run for each variable (n = 6; α′ = 0.008) and the total number of statistical tests run during the study (n = 109; α″ = 4.6×10^−4^). Herein, p-values that were significant with the Bonferroni corrections are indicated with a * symbol for α′ and a ** symbol for α″. All averaged data are reported herein as intersubject mean ± standard deviation.

**Figure 2:**
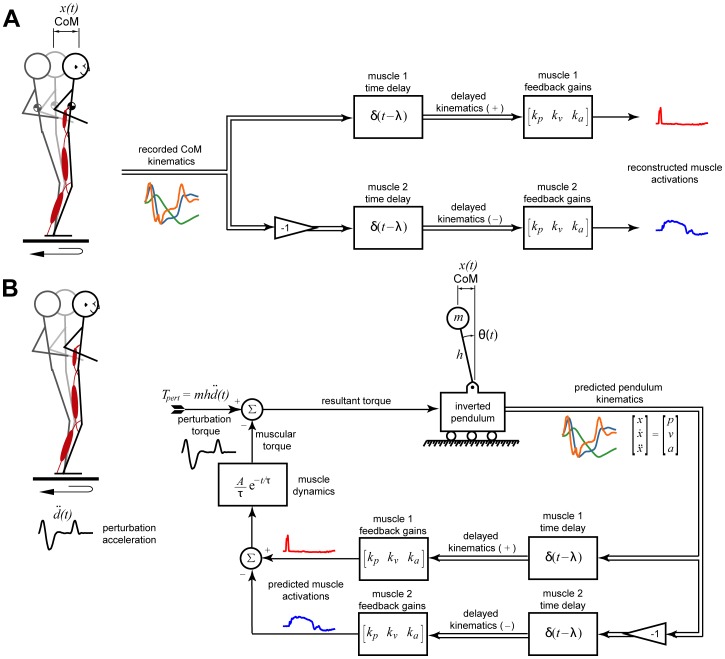
Feedback models for balance control. In the model formulation, an agonist muscle (muscle 1) responded to forward-directed kinematic signals only and its antagonist (muscle 2) responded to backward-directed kinematic feedback. **a)** Muscle activation patterns were characterized by feedback gains of the sensorimotor response model. Recorded EMG responses were reconstructed using delayed and weighted sum of recorded CoM kinematic signals (acceleration, velocity, and displacement). **b)** An inverted pendulum model of human balance was perturbed using torques calculated from experimentally-recorded platform motion and used to compute an optimal muscle response pattern. The horizontal kinematics (acceleration, velocity, and displacement) of the pendulum model were delayed, weighted, and summed to provide an EMG response. A first-order muscle model was then used to convert this model-derived muscle activity into a muscular torque to counteract the perturbation. The optimal solution was found by minimizing a tradeoff between reducing task-level errors versus muscle activation level.

To quantify whether changes in reactive muscle activity reflected central changes in the sensorimotor transformation and not just the reduced CoM kinematic deviations, we computed the relationship between measured EMG patterns to recorded CoM deviations using our sensorimotor response model [20], [22], [23]. Reactive EMG activity was reconstructed based on a weighted sum of recorded CoM displacement (*x_com_*), velocity (*v_com_*), and acceleration (*a_com_*) signals at a time delay:
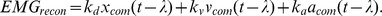



Thus, three feedback gains (or weights; *k_d_, k_v_, k_a_*) and a lumped time delay (*λ*) were chosen to minimize the error between the recorded EMG signals, which were averaged in blocks of 3 trials, and the reconstructed signals based on the sensorimotor response model (**Figure 2b**). The goodness-of-fit was evaluated using both the coefficient of determination (r^2^) and the uncentered coefficient of determination (variability accounted for; VAF) [43], [44]. As with the measured parameters, we performed a paired t-test on each feedback parameter (α = 0.05) to determine whether these feedback parameters exhibited significant differences within and across perturbation sets. We also fit the mean feedback parameters to an exponential function in each set.

Finally, for each subject, we compared reactive EMG signals to an optimal solution predicted from a simple neuromechanical model of balance [20], [22], [23]. A simulation of an inverted pendulum was scaled to the mass (*m*) and height (*h*) of each subject. The base of the pendulum was then subjected to the experimentally-recorded perturbation accelerations for forward and reversing perturbations [20], [22], [23]. The activation of a first-order muscle model acting about the base of the pendulum was defined by a delayed feedback rule whereby the predicted muscle activity was based on the simulated displacement (*x*), velocity (*v*), and acceleration (*a*) of the pendulum subject to a time delay (*λ*):
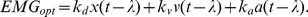



An optimal EMG solution then was found by selecting feedback gains (*k_d_, k_v_, k_a_*) and a lumped time delay that minimized a cost function consisting of a weighted sum-of-squared muscle activation (representing energy expenditure) and the sum-of-squared kinematic deviations of the pendulum from the upright configuration (representing task performance) (**Figure 2a**). For each subject, the RMS error of EMG averages across each block of 3 consecutive trials was compared to the optimal solution.

## Results

### Changes in CoM Kinematics Across Perturbation Sets

The patterns of task-level error quantified by CoM kinematics were consistent with adaptive changes, as large errors were encountered at the beginning of both the Reversal and Washout sets, which decreased over each set.

**Figure 3:**
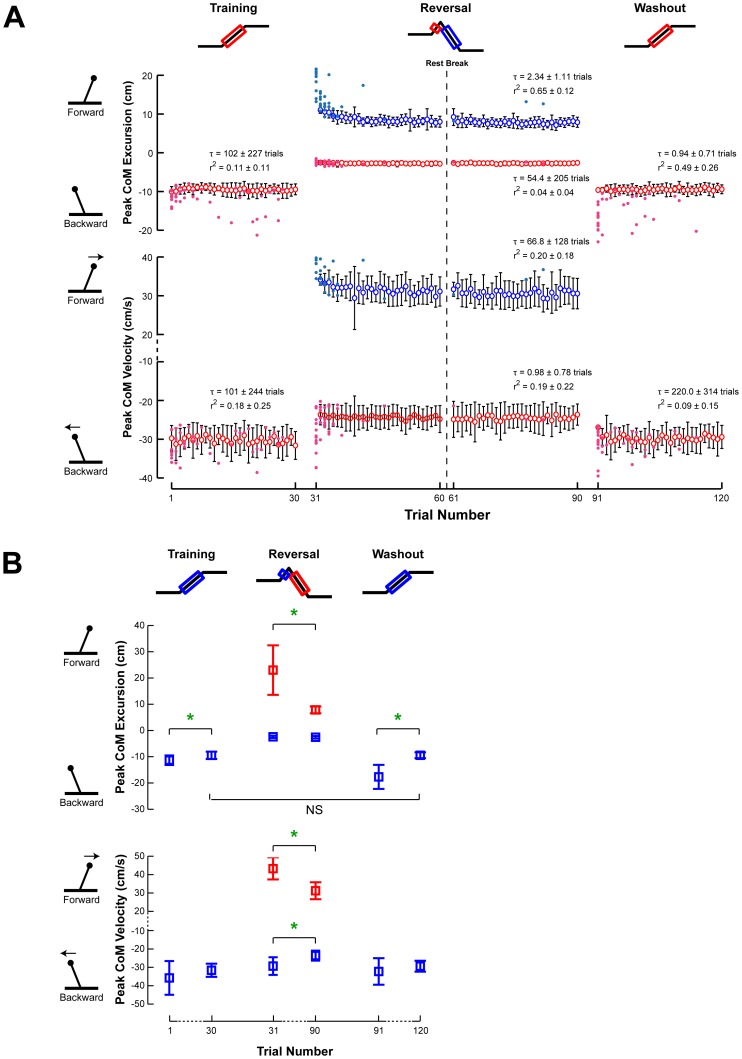
Changes in CoM kinematics across perturbation sets. **a)** The changes in mean peak CoM displacement and velocity for each perturbation set are illustrated with respect to trial number. Open circles and error bars represent the intersubject mean and standard deviation. On trials in which a subject took a step to recover their balance, the peak CoM displacement and velocity during the step is indicated with a filled circle. Red represents peak backward CoM kinematics in response to forward platform motion and blue represents forward CoM kinematics in response to backward platform motion. **b)** The mean peak CoM displacement and velocity for the first and last perturbations of each illustrate the data on which statistical comparisons performed. Parameters that changed significantly are indicated by a green star. NS is used to highlight certain insignificant comparisons between perturbation sets.

Although the very first trial of the Training set was the first time any of the subjects were exposed to the perturbation paradigm, the maximum backward CoM displacement and velocity was very consistent throughout the set, decreasing only slightly. In the Training set, the peak backward CoM displacement decreased from 11±2 cm to 9±1 cm (p = 0.004*) (**Figure 3**). A small decrease in peak CoM velocity was not significant (35±9 cm/s to 32±4 cm/s; p = 0.13). Throughout the Training set, subjects occasionally took steps, resulting in trials with large CoM displacements (**Figure 3**, colored dots).

On the first trial of the Reversal set, all subjects took a forward step due to the unanticipated reversal of the support surface direction from forward to backward after 100 ms. Maximum CoM displacement and velocity then decreased throughout the remainder of the set (**Figure 3**, Reversal). All subjects were able to withstand reversing perturbations without stepping within 5 trials. With successive perturbations, the steps became progressively shorter and slower until a non-stepping response was sufficient for subjects to maintain balance. The standing balance responses also changed qualitatively from a hip strategy to an ankle strategy response. In the Reversal set, the peak forward CoM displacement decreased from 23±9 to 8±1 cm (p<10^–16^**), including stepping trials, and peak forward CoM velocity decreased from 43±6 cm/s to 31±5 cm/s (p<10^–16^**) (**Figure 3**, Reversal, blue). Interestingly, a slight increase in peak forward CoM excursion (8±1 cm to 9±2 cm; p = 0.036) was observed following the rest period (administered at the mid-point of the Reversal set), demonstrating the effect of time on the adaptation process [45]. Although the initial forward portion of the reversing perturbation was identical across all perturbations, there were no significant changes in the peak backward CoM displacement (2±0.4 cm to 3±0.5 cm; p = 0.29), save a small decrease in peak CoM velocity from 29±5 cm/s to 24±3 cm/s (p<10^–4^**) (**Figure 3**, Reversal, red).

In contrast to the Training set, almost all subjects took a large backward step in the first trial of the Washout set, followed by a rapid decrease in CoM excursion (**Figure 3**, Washout). Peak CoM displacement decreased from 18±5 cm to 9±1 cm (p<10^–16^**), but no significant changes in peak CoM velocity were found (32±7 cm/s to 29±3 cm/s; p = 0.21). At the end of the Washout set, the peak backward CoM displacement was similar to that at the end of the Training set (p = 0.86), suggesting a complete washout of any adaptive changes made during the Reversal set.

### Changes in Anticipatory Postural Control Across Perturbation Sets

**Figure 4:**
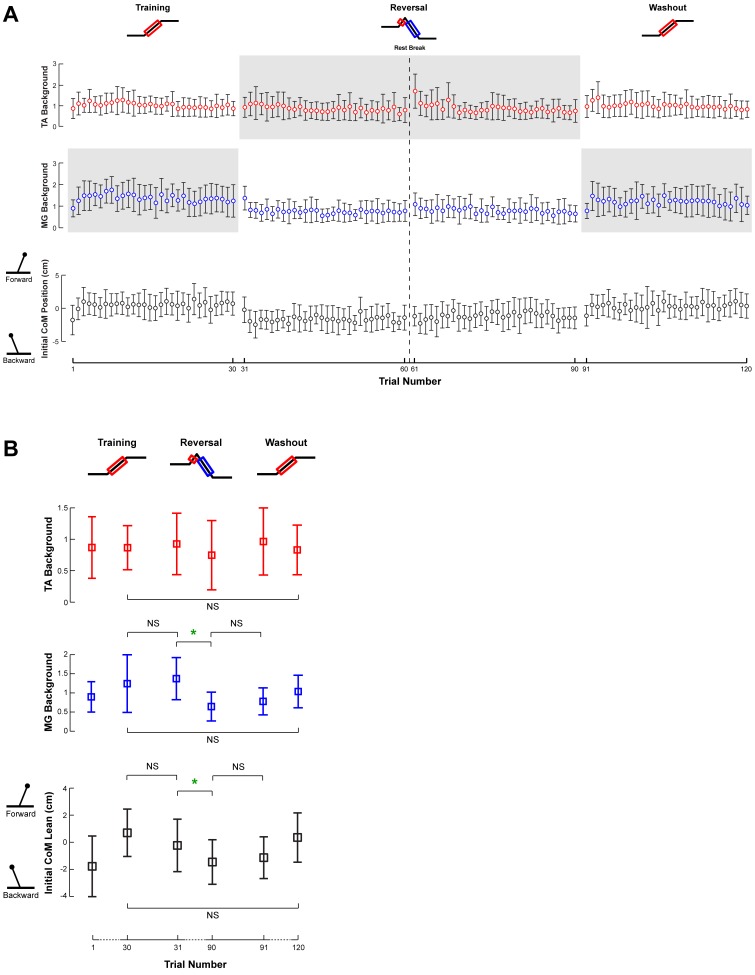
Feedforward changes in muscle activity and forward lean across perturbation sets. **a)** The changes in background TA and MG muscle activity, as well as initial subject lean, are illustrated with respect to trial number. Open circles and error bars represent the intersubject mean and standard deviation of the given parameter during each trial. Forward initial lean is indicated by positive values. Gray shaded areas indicate the adaptation of background muscle activity in antagonist muscles. **b)** Background muscle activity and initial subject lean for the first and last perturbations of each set illustrate data used for statistical comparisons. Parameters that changed significantly are indicated by a green star. NS is used to highlight certain insignificant comparisons between perturbation sets.

Only a few, relatively weak changes in postural tone and postural lean were found across perturbation sets, but they did exhibit aftereffects (**Figure 4**). The level of background TA activity did not change in any set, as TA is not typically necessary for quiet standing. No significant changes were found in the level of background MG activity or postural lean in the Training set. However, the background MG activity exhibited a nonsignificant trend of increasing by 43% (p = 0.10), consistent with a small forward shift in the initial postural lean from −1.78±2.24 cm to 0.10±2.02 cm (p = 0.63). Changes in background MG activity were identified in the Reversal set, during which MG decreased by 53% (p = 0.0035*), consistent with the backward shift of initial body lean from −0.23±1.94 cm to −3.89±6.45 cm (p = 0.04). Similar to the Training set, there were only trends but no statistically significant changes in background MG activity and postural lean in the Washout set. From one set to another, the background MG and initial lean in the first trial of one set were not significantly different from those in the last trial of the prior set (all p>0.05). Additionally, all anticipatory measures were similar at the end of the Training and Washout sets (p>0.21), suggesting a washout of the modest changes incurred during the Reversal set.

### Changes in Reactive Muscle Activity Across Perturbation Sets

We found adaptive changes in both the reactive muscle activity as well as the sensitivity of the sensorimotor response to perturbations.

**Figure 5:**
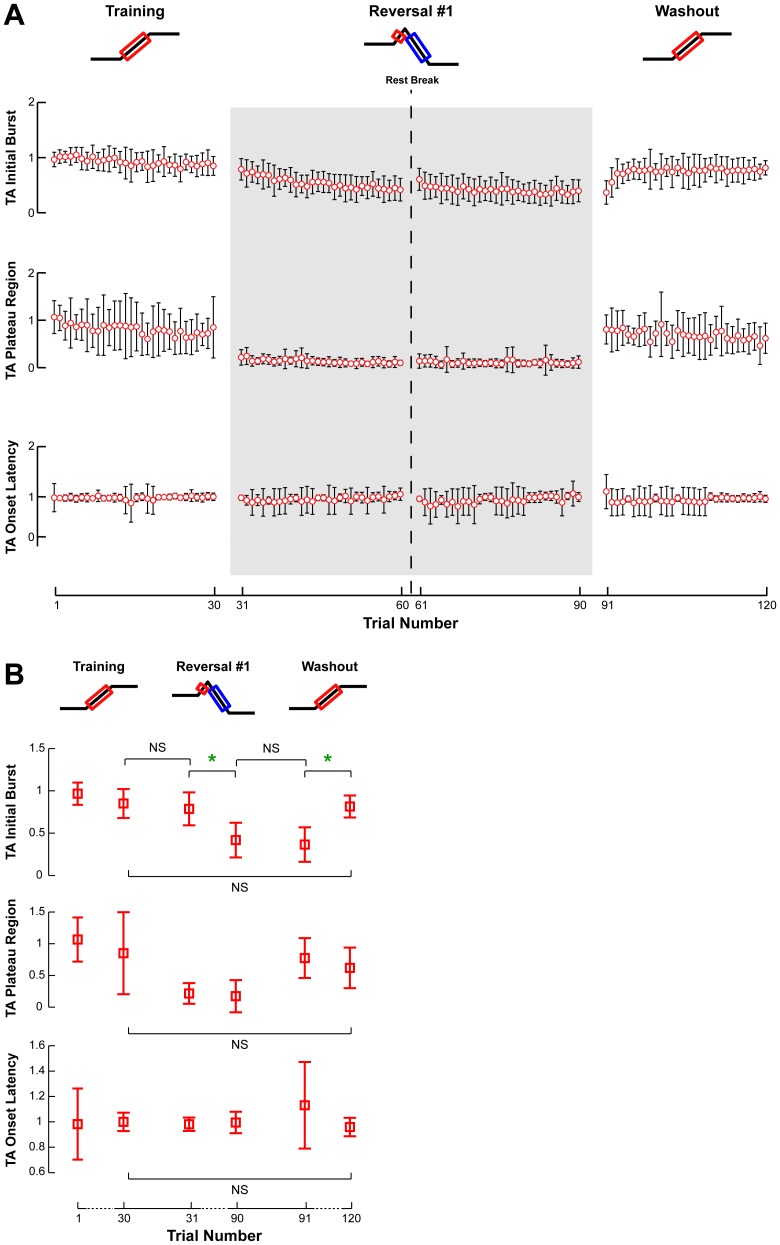
Changes in TA EMG responses across perturbation sets. **a)** The average amplitudes of recorded TA EMG patterns are illustrated with respect to trial number for muscle activity during the initial burst and plateau regions, as well as muscle onset latency. Open circles and error bars represent the intersubject mean and standard deviation. Gray shaded areas indicate the adaptation of antagonist muscle responses. **b)** The amplitudes of recorded TA EMG patterns for the first and last perturbations of each set illustrate data used for statistical comparisons. Parameters that changed significantly are indicated by a green star. NS is used to highlight certain insignificant comparisons between perturbation sets.

**Figure 6:**
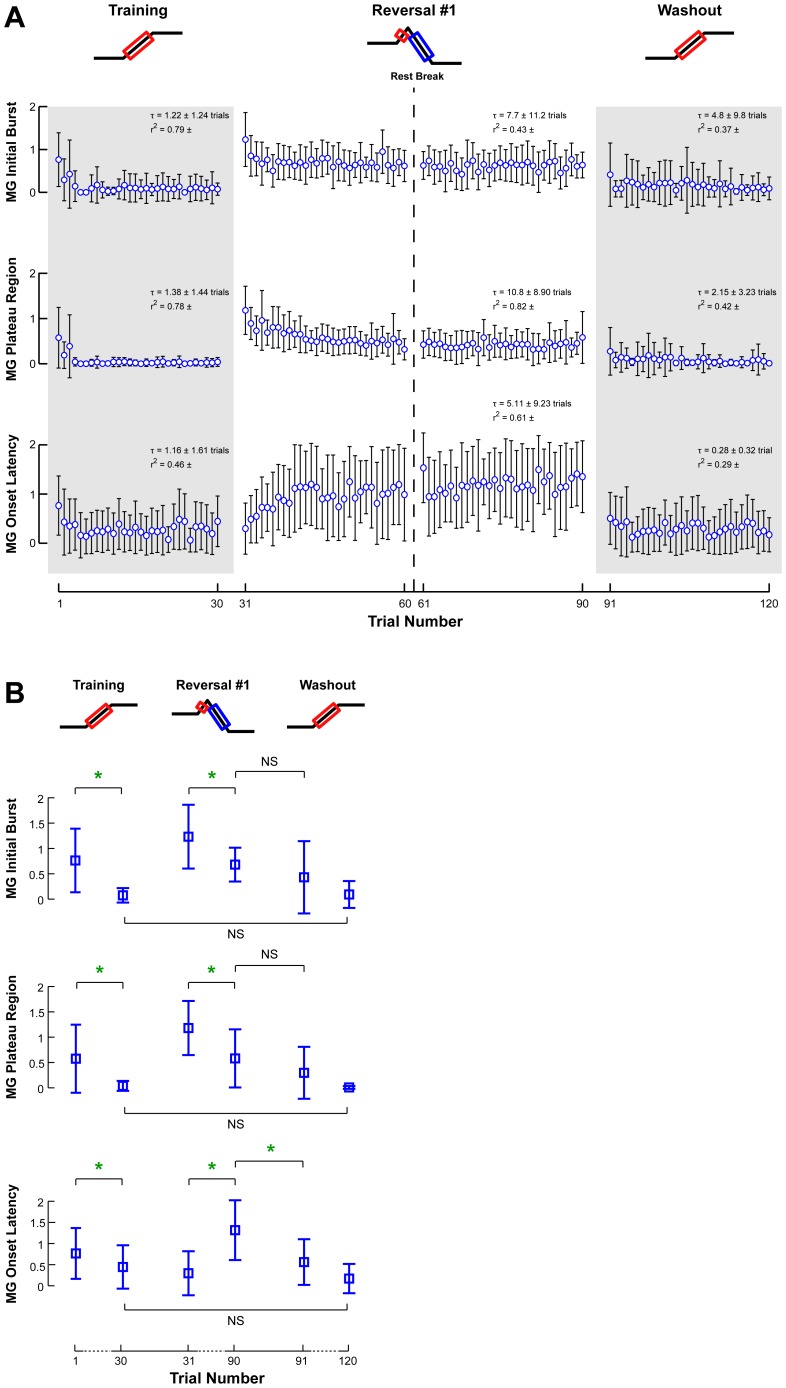
Changes in MG EMG responses across perturbations sets. **a)** The average amplitudes of recorded MG EMG patterns are illustrated with respect to trial number for muscle activity during the initial burst and plateau regions, as well as muscle onset latency. Open circles and error bars represent the intersubject mean and standard deviation. Gray shaded areas indicate the adaptation of antagonist muscle responses. **b)** The amplitudes of recorded MG EMG patterns for the first and last perturbations of each set illustrate data used for statistical comparisons. Parameters that changed significantly are indicated by a green star. NS is used to highlight certain insignificant comparisons between perturbation sets.

Despite the modest changes in CoM displacement and velocity in the Training set, the MG but not TA responses decreased across trials. In response to forward perturbations, muscular responses of the agonist muscle TA were observed at a latency of 116±3 ms across subjects. As the Training set progressed, there was no significant change in TA muscle activity in the initial burst (12%; p = 0.06) and or plateau regions (20%; p = 0.22) (**Figure 5**, Training). TA muscle activity was frequently accompanied by a dynamic co-contraction in antagonist MG, especially during the first few responses. Antagonist MG activity decreased by 90% (p = 0.0005*) and was essentially eliminated after one trial (**Figure 6**, Training). No changes in the onset latencies of either TA or MG were found.

In the Reversal set, reactive TA and MG muscle activity both decreased and MG onsets were delayed. Because of the short duration of the forward platform motion, TA exhibited a small initial burst and no plateau, which was followed by reactive MG activity at a latency of 186±20 ms with respect to the onset of negative platform acceleration. In this condition, the activity of the TA evoked by the initial forward platform motion is coincident with the reversal of the platform direction and therefore has a destabilizing, rather than stabilizing effect. The TA initial burst in the first trial of the Reversal set was not different from the last trial of the Training set (p = 0.22) and then decreased by 47% (p<10^−5^**) over the course of the Reversal set (**Figure 5**, Reversal). There was no change in TA onset latency across trials (p = 0.67). MG activity, which was necessary for balance, decreased in both the initial burst (48%; p = 0.002*) and plateau (51%; p = 0.04) (**Figure 6**, Reversal); MG onset latency did not change significantly, despite a trend toward longer onset delays (p = 0.08).

In the Washout set, TA activity increased and antagonist MG activity was quickly eliminated. The TA initial burst in the first trial of the Washout set was not significantly different from the last trial of the Reversal set (p = 0.88) and then increased 23% to a level that was not different from that at the end of the Training set (p = 0.57) (**Figure 5**, Washout). There was no significant change in the TA plateau region (p = 0.13). The MG initial burst and plateau regions had similar magnitudes in the first trial of the Washout set compared to the last trial of the Reversal set (p = 0.32 and p = 0.27, respectively) (**Figure 6**, Washout). This dynamic co-contraction demonstrated a trend of decreasing by 79% (p = 0.10). Interestingly, the MG onset latency in the first trial of the Washout set was shorter than the last trial of the Reversal set (84±78 sec vs. 195±75 sec; p = 0.0004), and did not change over the Washout set (p = 0.18).

**Figure 7:**
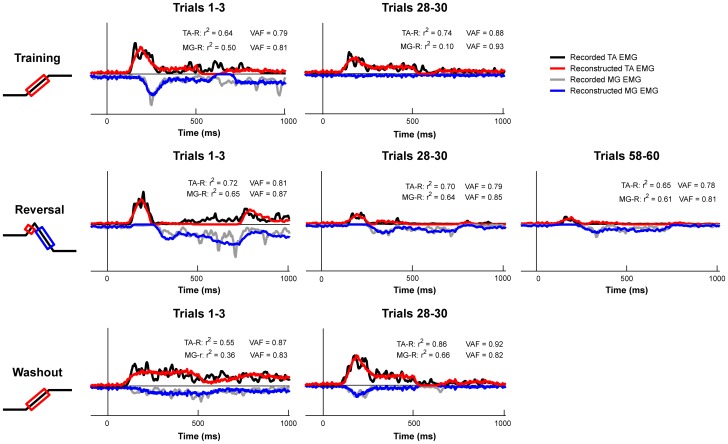
Feedback decomposition of EMG changes across perturbation sets. For a representative subject, recorded (black/gray) and reconstructed (red/blue) EMG signals are illustrated for Training, Reversal, and Washout sets. The goodness-of-fit between model-derived muscle activation patterns and recorded EMG is indicated by the coefficient of determination (r^2^) and the uncentered coefficient of determination (variability accounted for; VAF).

We were able to reconstruct the time course of muscle activity using weighted and delayed sums of CoM kinematics, demonstrating that the sensitivity of the reactive EMG decreased independent of the decreases in CoM excursion observed across perturbation sets (**Figure 7**). The sensorimotor response model accounted for 89±1% and 77±1% of the variability for TA and MG, respectively, in the Training set; 76±5% and 79±2% in the Reversal set; and 88±2% and 73±3% in the Washout set.

**Figure 8:**
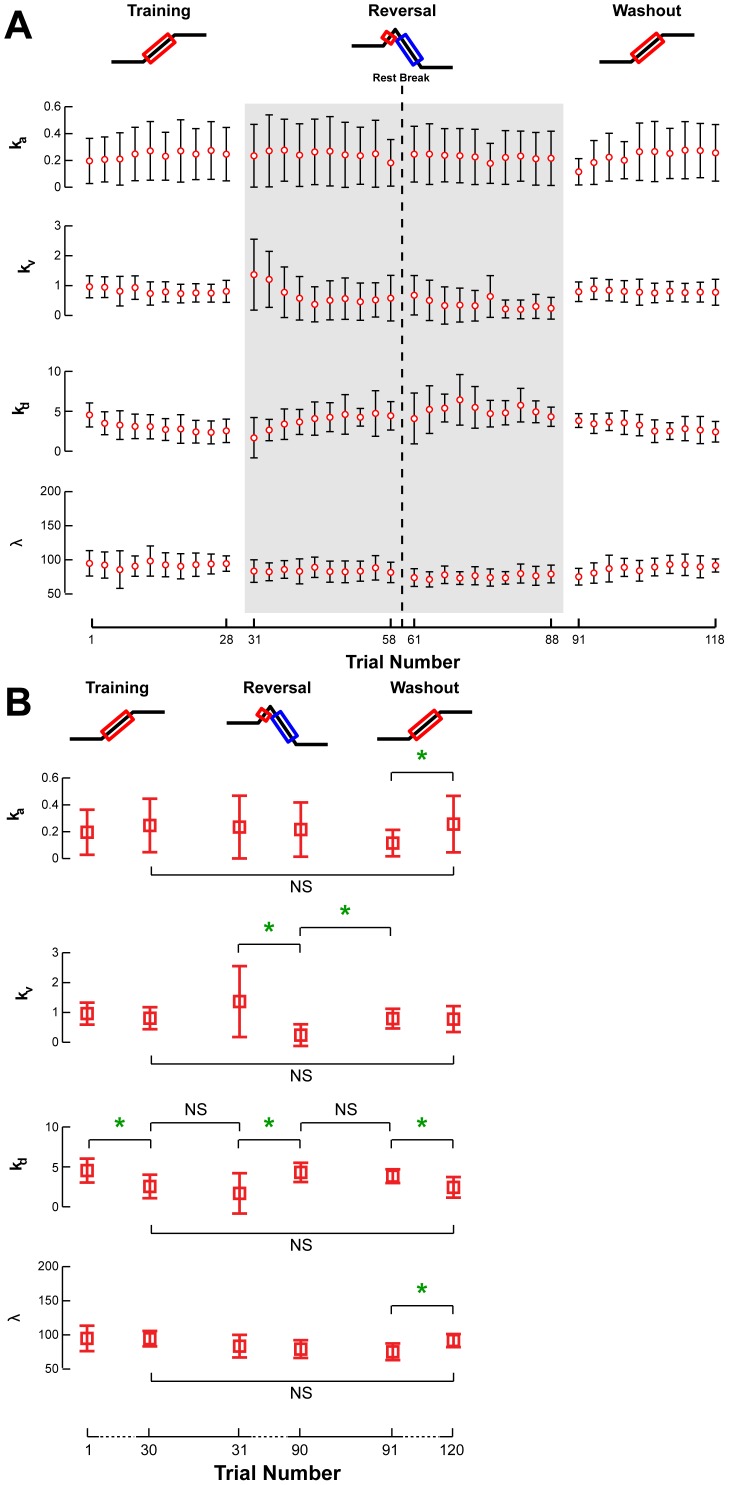
Changes in TA feedback gains across perturbation sets. **a)** The acceleration (*k_a_*), velocity (*k_v_*), and displacement (*k_d_*) gains, along with the time delay (*λ*), were averaged across all subjects and are illustrated for TA with respect to trial number. Open circles and error bars represent the intersubject mean and standard deviation. Gray shaded areas indicate the adaptation of feedback parameters for antagonist muscles. **b)** Average TA feedback gains for the first and last perturbations of each set are illustrated for statistical comparison. Parameters that changed significantly are indicated by a green star. NS is used to highlight certain insignificant comparisons between perturbation sets.

**Figure 9:**
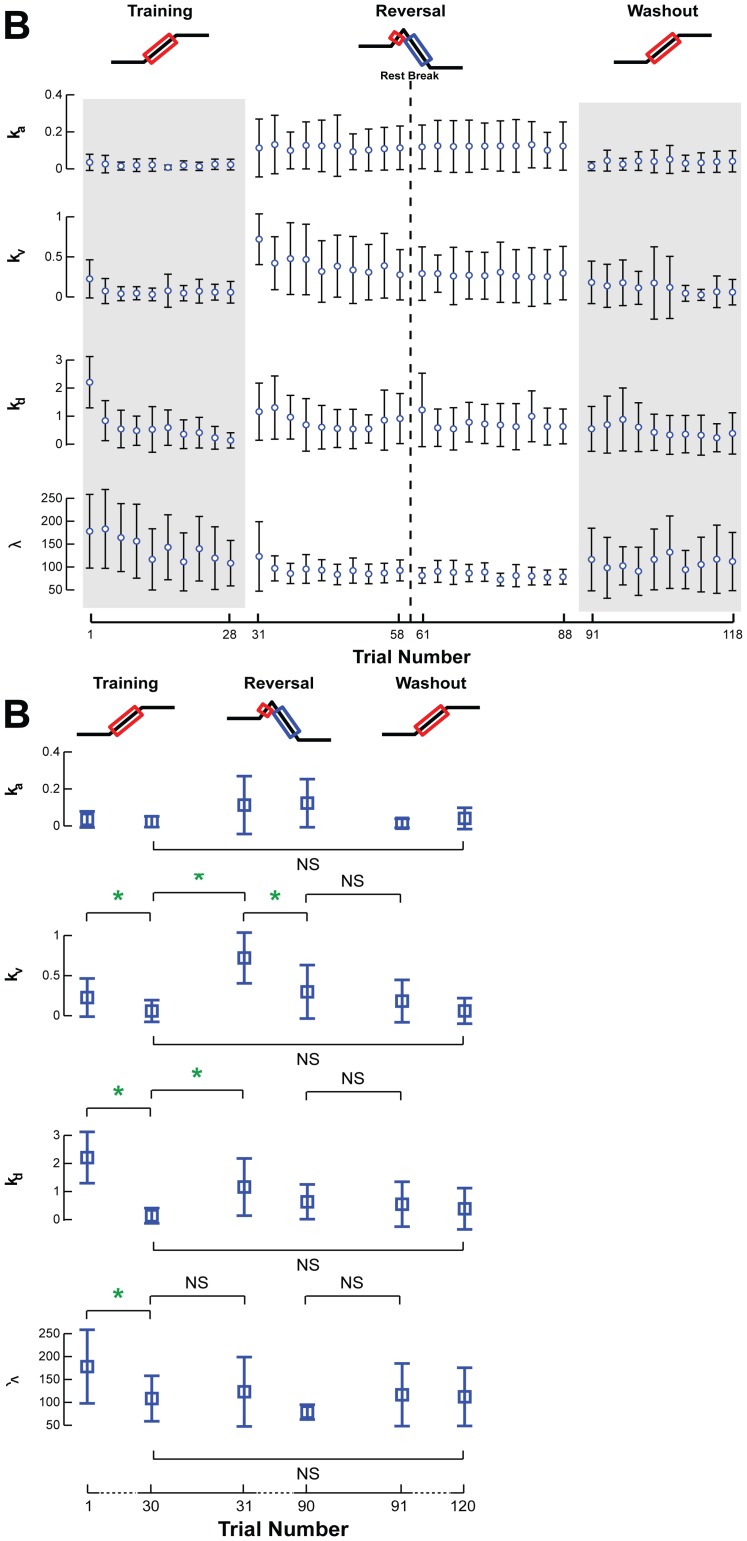
The adaptation of MG feedback gains across each perturbation set. The acceleration (*k_a_*), velocity (*k_v_*), and displacement (*k_d_*) gains, along with the time delay (*λ*), were averaged across all subjects and are illustrated for MG with respect to trial number. Open circles and error bars represent the intersubject mean and standard deviation. Gray shaded areas indicate the adaptation of feedback parameters for antagonist muscles. **b)** Average MG feedback gains for the first and last perturbations of each set illustrate data used for statistical comparisons. Parameters that changed significantly are indicated by a green star. NS is used to highlight certain insignificant comparisons between perturbation sets.

Adaptive changes in the sensorimotor response were also identified. In the Training set, TA velocity gain, *k_v_* (p = 0.15), and displacement gain, *k_d_* (p = 0.0002) showed decreasing trends (**Figure 8**, Training), and antagonist MG velocity (p = 0.009) and displacement gains (p<10^−6^**) decreased to zero (p>0.07) (**Figure 9**, Training**)**. The changes in the TA velocity and displacement gains were maintained between the last trial of the Training set and the first trial of the Reversal set (p>0.06). During the Reversal set, the TA, which was rapidly shut down, exhibited a decrease in velocity gain (p = 0.002*) and an increase in displacement gain (p = 0.013) (**Figure 8**, Reversal). MG velocity gain (p = 0.004*) decreased, and displacement gain had a decreasing trend (p = 0.06) that was not statistically significant (**Figure 9**, Reversal). TA displacement gain and MG velocity and displacement gains were not different between the last trial of the Reversal set and the first trial of the Washout set (p>0.39). Over the course of the Washout set, TA acceleration feedback gain, *k_a_*, increased (p = 0.006*), while displacement gain decreased (p = 0.009) (**Figure 8**, Washout). The latency or delay, *λ,* was constant for TA across all sets (p>0.75), save for an increase during the Washout set (p = 0.001*). Similarly, the delay for MG was constant over all sets (p>0.067), except a decrease during the Training set (p = 0.01). No other feedback gains for MG exhibited significant changes during the Washout set (**Figure 9**, Washout). At the end of the Washout set, all feedback gains for both TA and MG were similar to that at the end of the Training set (**Figures 8b** **and** **9b**; all p>0.11), suggesting that the washout of adaptive changes during the Reversal set also extended to the feedback gains.

**Figure 10:**
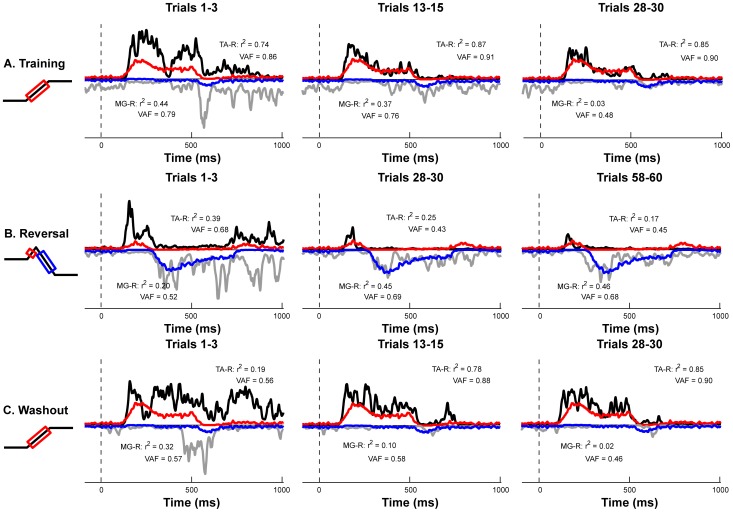
Changes in EMG activity compared to optimal motor pattern. For a representative subject, recorded (black/gray) and optimal (red/blue) TA and MG activity during early, middle, and late adaptation are illustrated for the **a)** Training, **b)** Reversal, and **c)** Washout sets. The goodness-of-fit between model-derived muscle activation patterns and recorded EMG is indicated by the coefficient of determination (r^2^) and the uncentered coefficient of determination (variability accounted for; VAF). Note that the optimal motor pattern remains constant with repetition of the perturbation, while recorded muscle activity adapts toward the optimal solution.

**Figure 11:**
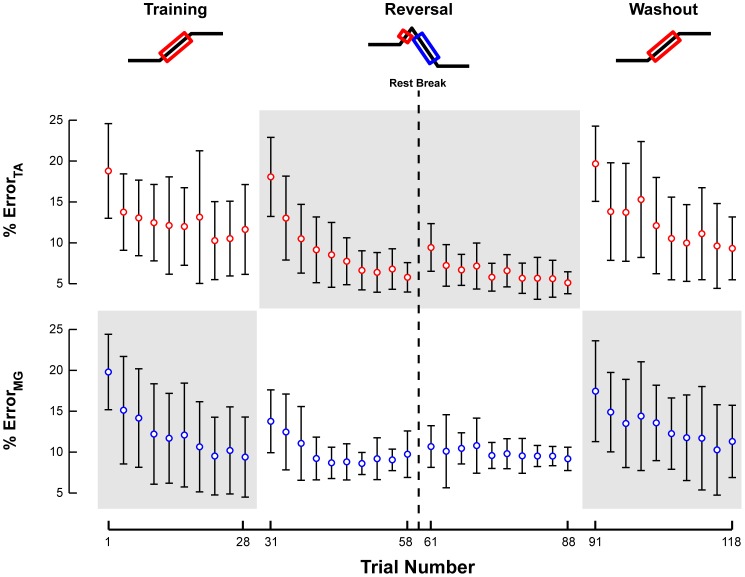
Reduction of error between recorded and optimal motor patterns. The average percent error between recorded TA and MG EMG patterns and the optimal motor solution derived from the inverted pendulum model is illustrated with respect to trial number. Error bars indicate one standard deviation of the mean percent error for three consecutive trials. Gray shaded areas indicate the adaptation of antagonist muscle responses.

Finally, the error between the reactive EMG activity and an optimal motor solution (**Figure 10**) decreased monotonically over each perturbation set (**Figure 11**). With repetition, the recorded muscle responses adapted toward the optimal motor solution for both TA and MG, with only 5 to 11% error by the end of each perturbation set for each muscle. The optimal solution did not predict dynamic co-contraction of MG in the Training and Washout sets. Accordingly, the error between the recorded and predicted MG muscle activity decreased significantly in all conditions (Training and Reversal: p<10^−4^**; Washout: p = 0.0011*) as this co-contraction was progressively eliminated from subject responses (**Figure 6a**).

## Discussion

We present evidence for motor adaptation primarily in the reactive sensorimotor response to perturbations during standing balance. This corroborates finding from the upper limb demonstrating that trial by trial modifications in long-latency responses to perturbations are generated by updating an internal model [46], [47]. Our work provides a mechanism to explain prior studies demonstrating that the amplitude of responses decreases with serial presentation of perturbations [27], [28], [32], which has been attributed to cerebellar adaptation or habituation. Here, motor adaptation was reflected in task-level movement error and in the sensitivity of the long-latency sensorimotor response where large aftereffects were induced by exposure to a novel perturbation. The aftereffect and reduction in error were not specific to the movement strategy, but task-level performance errors were monotonically reduced as the response transitioned from a stepping strategy to a hip strategy to an ankle strategy. This suggests that the magnitude of error experienced in prior perturbation trials is used to modulate the sensitivity of the sensorimotor response in subsequent trials. The idea that a common neural mechanisms underlies these different kinematic responses is consistent with our recent findings that task-level deviations of CoM kinematics from the desired, upright state–and not deviations of joint angles–govern muscle activity in reactive balance (Safavynia and Ting 2013), and can equally account for the initial muscle activity evoked in hip, ankle, and reactive stepping responses (Welch and Ting 2009; Chvatal et al. 2010). Because the reduction in muscle activity within each set was greater than that expected due to a proportional relationship between the induced error and the reactive muscle activity, they reflected adaptive changes in the sensitivity of the central sensorimotor response to error during reactive balance.

As in reaching adaptation experiments, we examined discrete events or trials, which allowed for the dissociation of feedforward (anticipatory) and feedback (reactive) components contributing to perturbed balance control. Feedforward components, such as postural lean and postural tone, are modified and executed prior to the perturbation onset and therefore must reflect a prediction based on prior trials. These feedforward components act to influence the initial stability of the subject, prior to any error accumulation or the triggering of reactive responses to that error. Similarly, the sensorimotor feedback response was also altered in a manner reflecting a prediction based on prior trials, but could only be observed in response to a sensory error. This nomenclature is in contrast to that used in split-belt locomotor adaptation, where the term “feedforward” has been used to identify features of gait that exhibit adaptation from stride to stride and “feedback” refers to features that do not change between strides [48], [49]. This difference in terminology is required due to the continuous nature of locomotion, where anticipatory adjustments to the motor control strategy could occur concurrently with delayed reactive responses to sensory information received during the previous phase of movement.

However, in contrast to most motor adaptation studies of voluntary movement, we identified either no or only modest changes in feedforward or anticipatory components such as background muscle activity or postural lean in the initial trials of each set. In balance control, changes in postural lean and postural tone may mitigate the effects of a perturbation by changing the mechanical response of the body to perturbation [50], reducing the required amplitude of the sensorimotor response [2]. This might be considered analogous to increased co-contraction during reaching in unstable environments, which may be controlled independently of initial reach direction [36], [51]. Here, we showed that anticipatory changes in postural lean and postural tone rapidly saturate in one or two trials. For postural lean, this saturation may occur due to biomechanical limits on the amount of lean that is possible before crossing the boundary of stability [52], [53], or due to increased energetic expenditure of standing quietly with more muscle activity for relatively long periods of time in relation to the perturbation duration (20 to 30 s between trials versus ∼500 ms per trial). Increased co-contraction may also decrease the relative maneuverability of the body. The small changes in postural lean and postural tone were consistent with adaptation and demonstrated aftereffects from one perturbation set to the next, suggesting that they may be modified by an internal model for reactive balance. However, as the changes in these anticipatory components were relatively modest and quick compared to the changes observed in sensorimotor response parameters, they could not explain the complete, extended reduction in CoM kinematics and muscle activity evoked by perturbations at the end of each set.

Changes in the sensitivity of the sensorimotor response to perturbation reflect predictive changes in the “central set” or internal model for reactive balance control. During reaching movements in a novel force field environment, adaptive changes in the initial reach direction are observed from trial to trial, reflecting updates to an internal model used for planning movement. Similarly, we demonstrate the sensorimotor response to balance perturbations is modified from trial to trial, which likely reflects stored changes in an internal model use to set the sensitivity of the response to sensory errors [46], [47]. This is analogous to the previously-described concept of “central set” [54], [55], in which the feedforward modification of the sensitivity of the nervous system to errors prior to a perturbation determines the magnitude of the evoked responses. Postural adjustments to sensory perturbations have demonstrated similar decrease in sensitivity over time [56]. Therefore the sensorimotor response to perturbation can be modified in a predictive manner [57], exhibiting gradually decreased magnitude as well as aftereffects.

Surprisingly, we observed a smooth transition of CoM kinematics across different postural behaviors during adaptation, consistent with the idea that the CoM is an important control variable for balance [1], [20], [22], [23], [26]. All subjects took a reactive step on the first trial of the Reversal set, however no obvious discontinuities in the adaptation of CoM displacement or velocity were observed, even as subjects progressively changed from a stepping response to a hip strategy response to an ankle strategy response. A monotonic decrease in CoM kinematics during the Reversal set could be described as an exponential decay (τ = 2.3±1.1 trials) in all subjects, but less so in other conditions. The smooth decline suggests that CoM kinematics are controlled during reactive balance independent of the particular joint motions used to achieve that control [21], [24], [58]. This is consistent with our findings that muscle activity could be equally reproduced using the same sensorimotor response model whether subjects used a hip, ankle, or mixed strategy (Welch and Ting 2009). Similarly, we showed that the initial response to perturbation in multiple muscles had a similar pattern but simply larger amplitude when subjects took a step or kept their feet in place to maintain balance (Chvatal et al 2011). Similarly, in three-dimensional reaching tasks, the same hand position can be achieved by a variety of joint angles [59], and it is well known for repeated motions that the endpoint trajectory of the arm is much more consistent than the individual joint motions [58], [60], [61]. In contrast, subjects occasionally took steps during Training and Washout sets that resulted in large discontinuities in CoM kinematics. We speculate that these intermittent large errors could have been part of a failed exploration process or they could have resulted from inattention or other sources.

We also found that there were limits to the extent that the sensorimotor feedback responses could be modified. Although the TA response to the initial forward direction of reversing perturbations was destabilizing, this response was not completely eliminated during the Reversal set. This is consistent with the observation that the automatic postural response is not voluntarily controlled and can only be modified in amplitude, but not completely muted [1], [55]. Because of the consistent timing of the reversing of platform motion during the Reversal set, it could be expected that MG activity would be elicited earlier, in anticipation of the perturbation reversal, with repeated exposure. However, there was a trend toward the MG responses being evoked later in time as the Reversal set progressed. This suggests that, instead of trying to more rapidly restore vertical equilibrium through early muscle activation, subjects appeared to take advantage of the reversing motion of the platform to passively return to the vertical, when possible. Interestingly, for more than half of the subjects, the MG was activated in the first few forward perturbations of the Washout set at the approximate timing as observed in the Reversal set, suggesting that there was some anticipatory component to the response. This aftereffect was progressively adapted out of the response strategy over the remainder of the Washout set.

Consistent with prioritizing stability over energetics, we observed substantial muscle co-contraction during the sensorimotor response to perturbations in early adaptation. However, as subjects improved their performance, muscle co-contraction decreased. Although CoM displacement decreased monotonically in the Reversal set, the underlying changes in muscle activity were highly variable from trial to trial and between subjects. We identified a significant decrease in muscle activity from the beginning to the end of the Reversal set and from the beginning to the end of the Washout set in TA, but we could not reasonably use an exponential decay to describe the changes in muscle activity. However, through visual inspection, it is clear that the CoM displacement decreased rapidly over the first few trials, whereas muscle activity and the sensitivity of the response changed over a longer time course. Despite inter-subject and inter-trial variability, as well as differences in the adaptive behaviors exhibited by each subject, the error between subject responses and the optimal solution was reduced with repetitive trials during each perturbation set. While not conclusive, these observations suggest that subjects continued to decrease energetic expenditure as they became more familiar with the perturbations even if task performance did not improve. Similarly, a decrease in the metabolic cost and muscle activity in reaching has been demonstrated during the adaptation of voluntary arm movements [38] and walking [39]. Taken together, is possible that adaptation of the internal model for balance is driven by a constantly-evolving tradeoff between stability and energetic expenditure [51], [62] based on prior experience.
